# Supplementary material for: Distinct kinetics of antibodies to 111 Plasmodium falciparum proteins identifies markers of recent malaria exposure
Source: Nat Commun. 2022 Jan 17;13:331. doi: 10.1038/s41467-021-27863-8 (PMC8764098; doi:10.1038/s41467-021-27863-8)
Supplement: Supplementary file 1 — Supplementary Information [file 41467_2021_27863_MOESM1_ESM.pdf]

Distinct kinetics of antibodies to 111 *Plasmodium falciparum* proteins identifies markers of recent malaria exposure

Yman V, Tuju J, White M T, Kamuyu G, Mwai K, Kibinge N, Asghar M, Sundling C, Sondén K, Murungi L, Kiboi D, Kimathi R, Chege T, Chepsat E, Kiyuka P, Nyamako L, Osier F H A, Färnert A

Supplementary Information

Supplementary Figures

Supplementary Fig. 1.

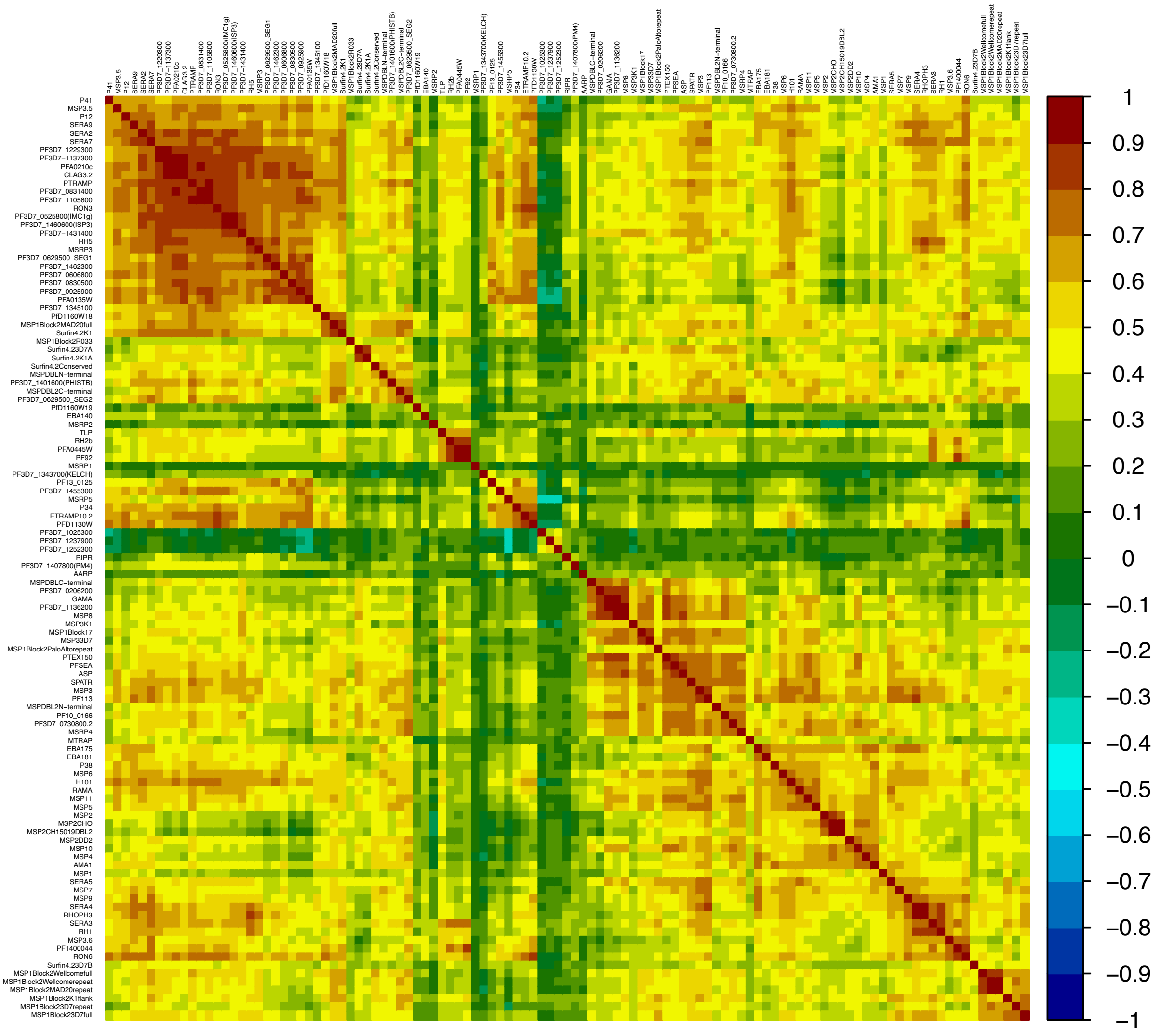

Heatmap displaying the correlation of all pairwise comparisons of antibody responses (n = 6105). Positive correlation is indicated in red while negative correlation is indicated in blue. The magnitude of the correlation is given by the colour intensity (-1: blue, 0: green, 1: darkred). Responses are ordered using a hierarchical clustering based on the magnitude of the correlation.

Supplementary Fig. 2

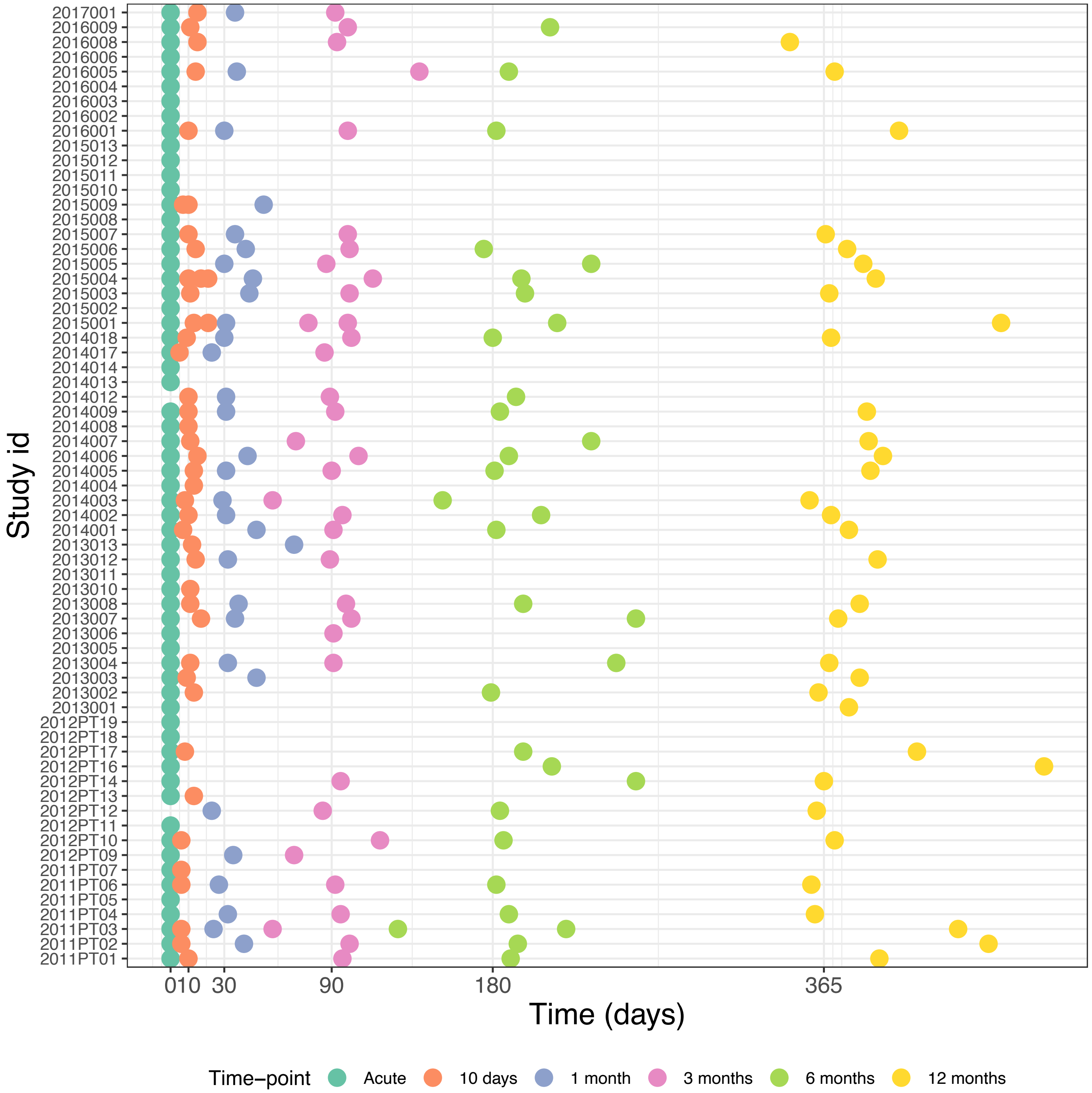

Scatterplot detailing the follow-up of study participants. Each row corresponds to an individual study participant. Dots represent the time-point in days after enrolment at which the individual was sampled. Colours denote the intended follow-up scheme with sampling at the acute time-point (green) and after 10 days (red), 1 month (blue), 3 months (pink), 6 months (green), and 12 months (yellow).

Supplementary Fig. 3

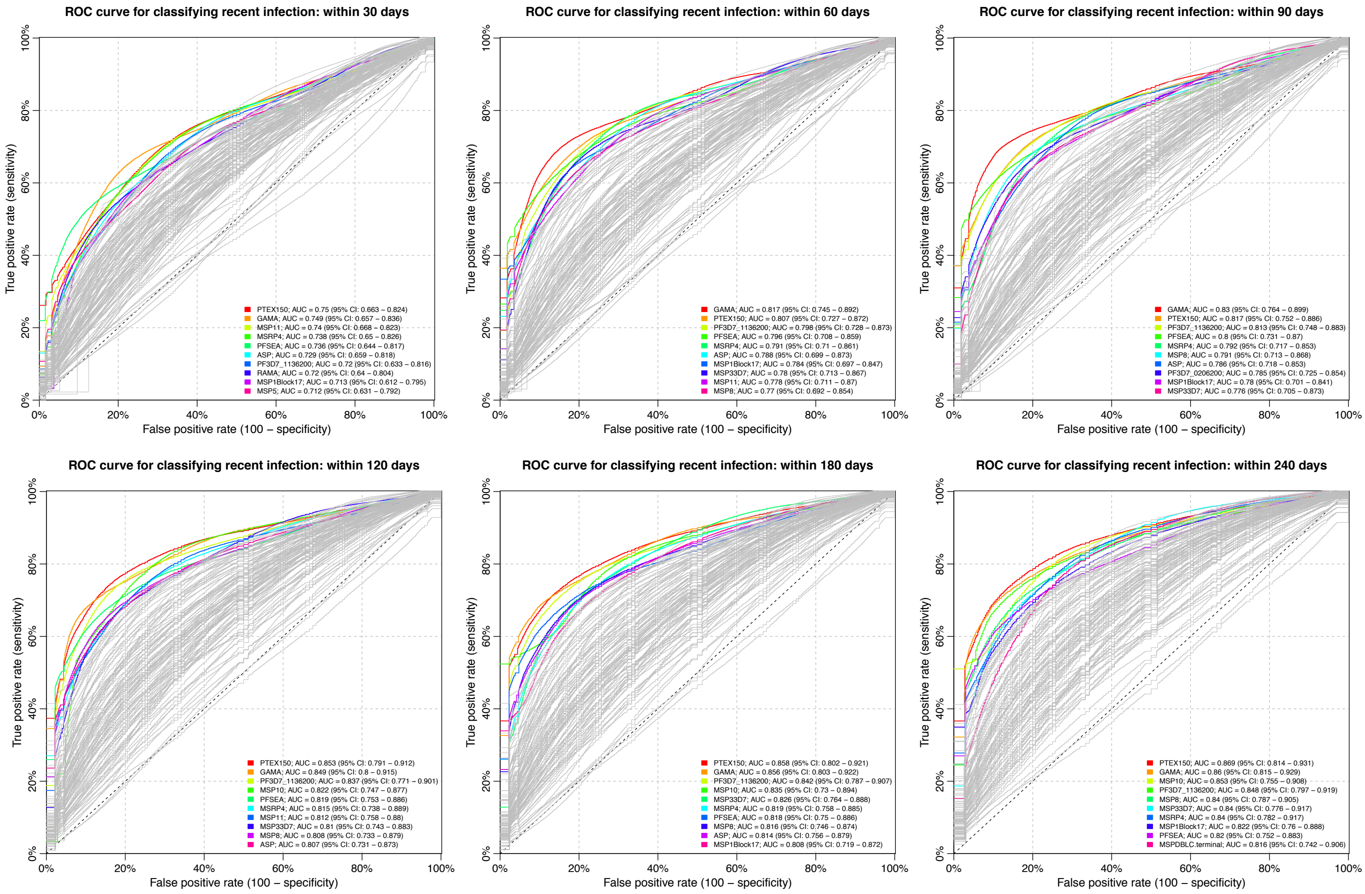

Receiver operating characteristic (ROC) curve for classifying individuals as recently infected using a threshold antibody level to a single antigen. The analysis was repeated for a range of temporal thresholds used to define a recent infection (i.e. 1, 2, 3, 4, 6, and 8 months) and each panel represents the results for a given temporal threshold. Coloured curves in each panel correspond to the top 10 antibody responses that were most predictive of recent infection as determined by the classifier area under the ROC curve (AUC). Rainbow coloured lines correspond to the ten classifiers with the highest cross-validated AUCs.

Supplementary Fig. 4

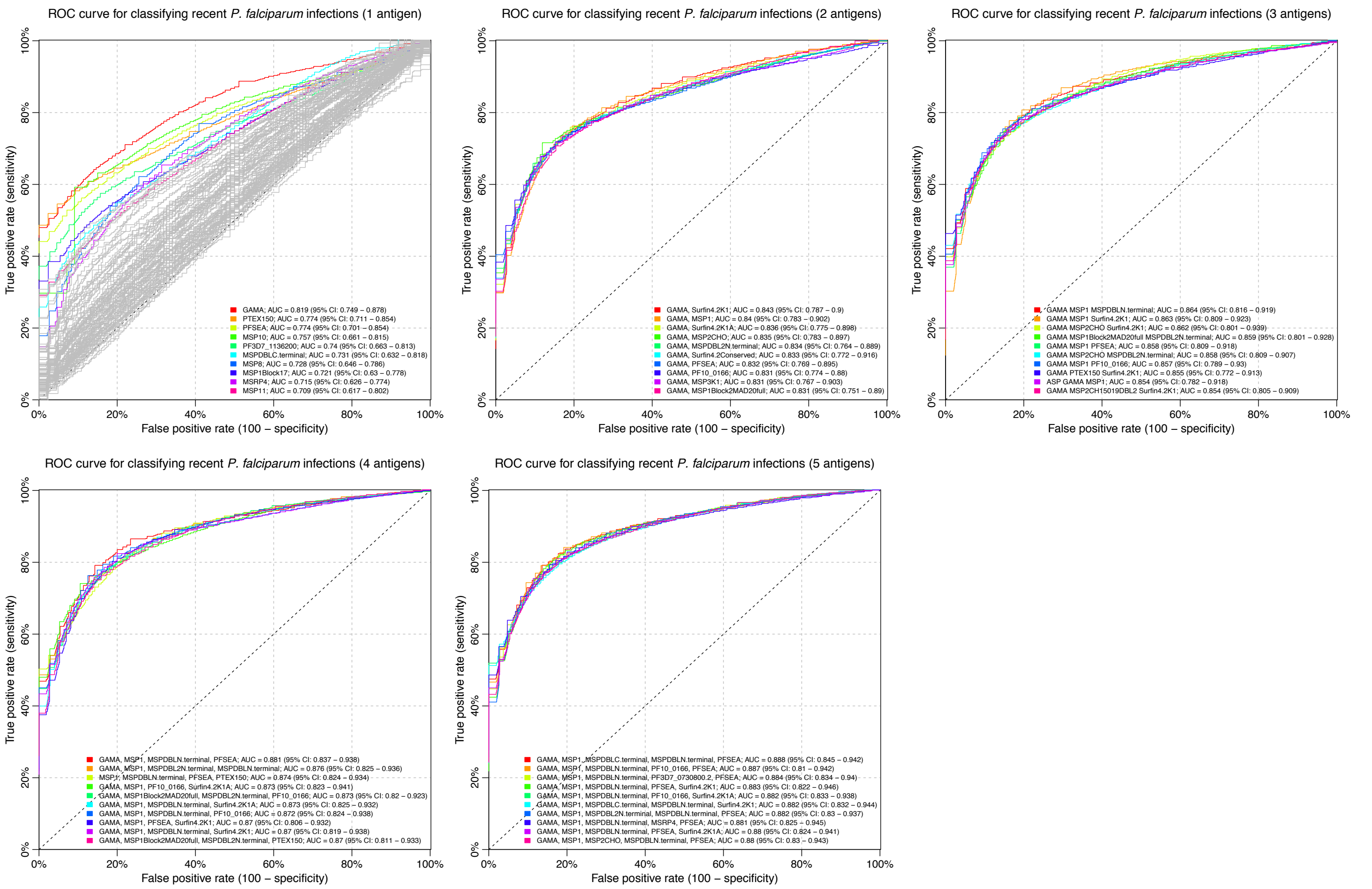

Cross-validated ROC-curves of classifier performance for random forest classifiers fitted to data on antibody responses towards a combination of two to five out of the 28 selected antigens. There was a gradual increase in random forest classifier performance with the inclusion of increasing number (1 to 5) of antibody responses. Individual panels represent the cross-validated receiver operating characteristic (ROC) curves for random forest classifiers fitted to data on antibody responses to the top 10 combinations of one (111), two (379 combinations), three (3276 combinations), four (20475 combinations), and five (98280 combinations) antibody responses out of the 28 selected, respectively. The classifier performance was evaluated using the area under the ROC curve (AUC). Rainbow coloured lines correspond to the ten classifiers with the highest cross-validated AUCs.

Supplementary Fig 5

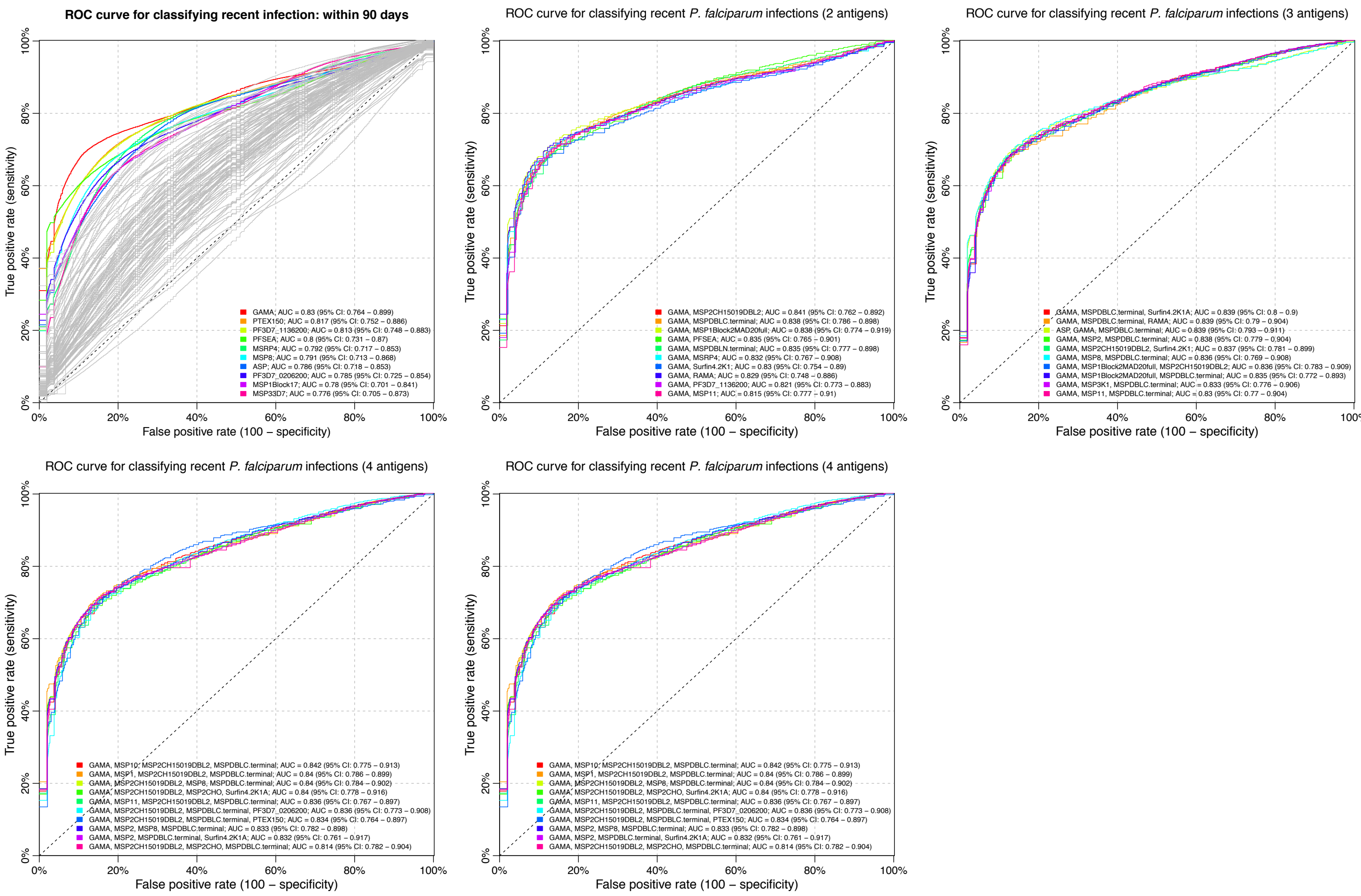

Cross-validated ROC-curves of classifier performance for logistic regression classifiers fitted to data on antibody responses towards a combination of two to five out of the 28 selected antigens. There was no significant increase in logistic regression classifier performance with the inclusion of increasing number (1 to 5) of antibody responses. Individual panels represent the cross-validated receiver operating characteristic (ROC) curves for logistic regression classifiers fitted to data on antibody responses to the top 10 combinations of one (111), two (379 combinations), three (3276 combinations), four (20475 combinations), and five (98280 combinations) antibody responses out of the 28 selected, respectively. The classifier performance was evaluated using the area under the ROC curve (AUC). Rainbow coloured lines correspond to the ten classifiers with the highest cross-validated AUCs.

Supplementary Fig 6

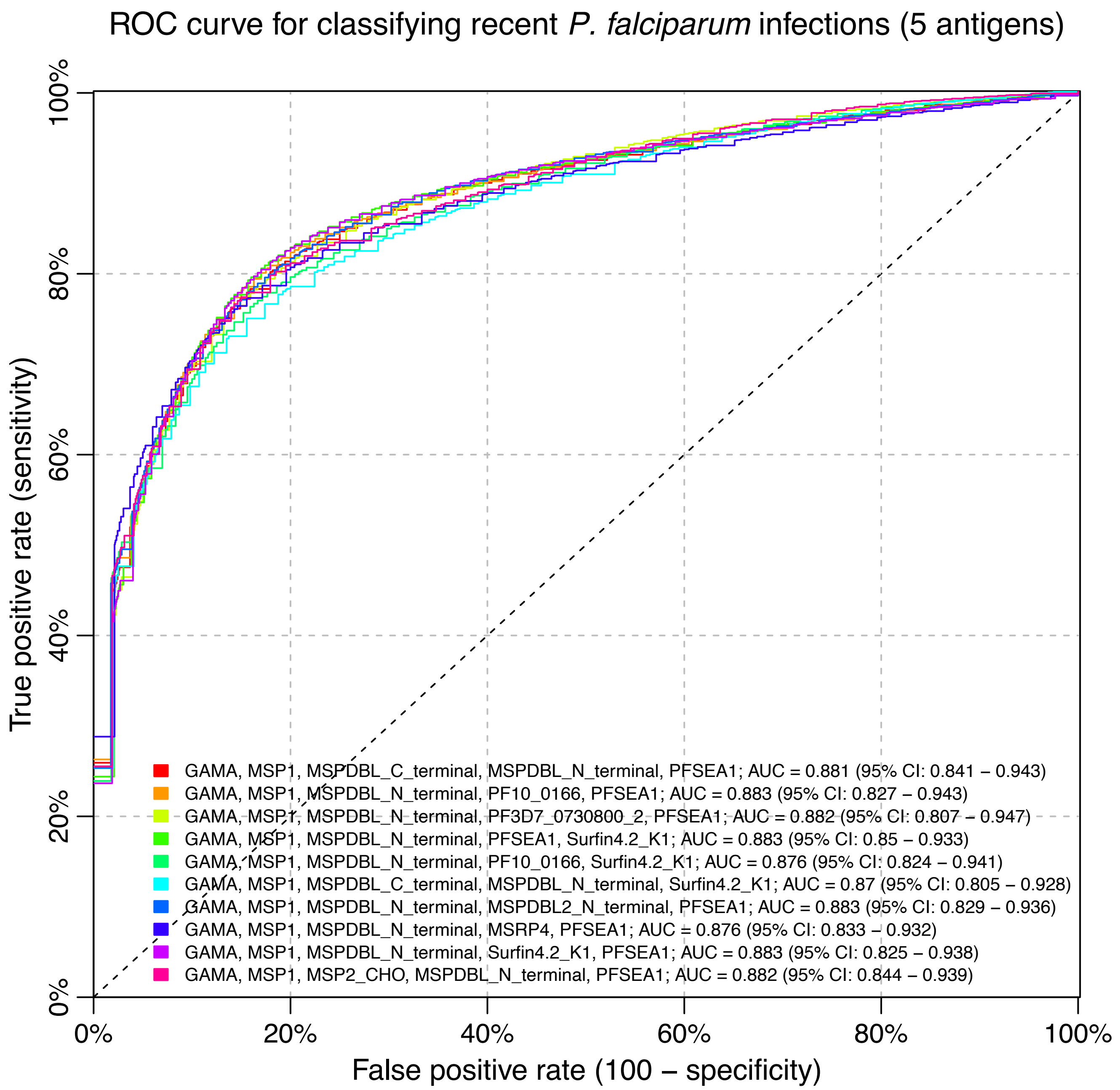

Cross-validated ROC-curves of classifier performance for random forest classifiers fitted to data on antibody responses to the best combination of five out of the 28 selected antigens. The underlying analysis utilises an alternative method for cross-validation which ensures that the same individual is not represented in both the training and the test set. This alternative approach had no impact on the classifier performance as indicated by the AUC which is highly similar to the AUC obtained using the main approach (see Figure 4B and/or Supplementary Fig S4). Rainbow coloured lines represent the individual ROC curves for random forest classifiers fitted to different combinations of antibody redsponses.

Supplementary Fig 7

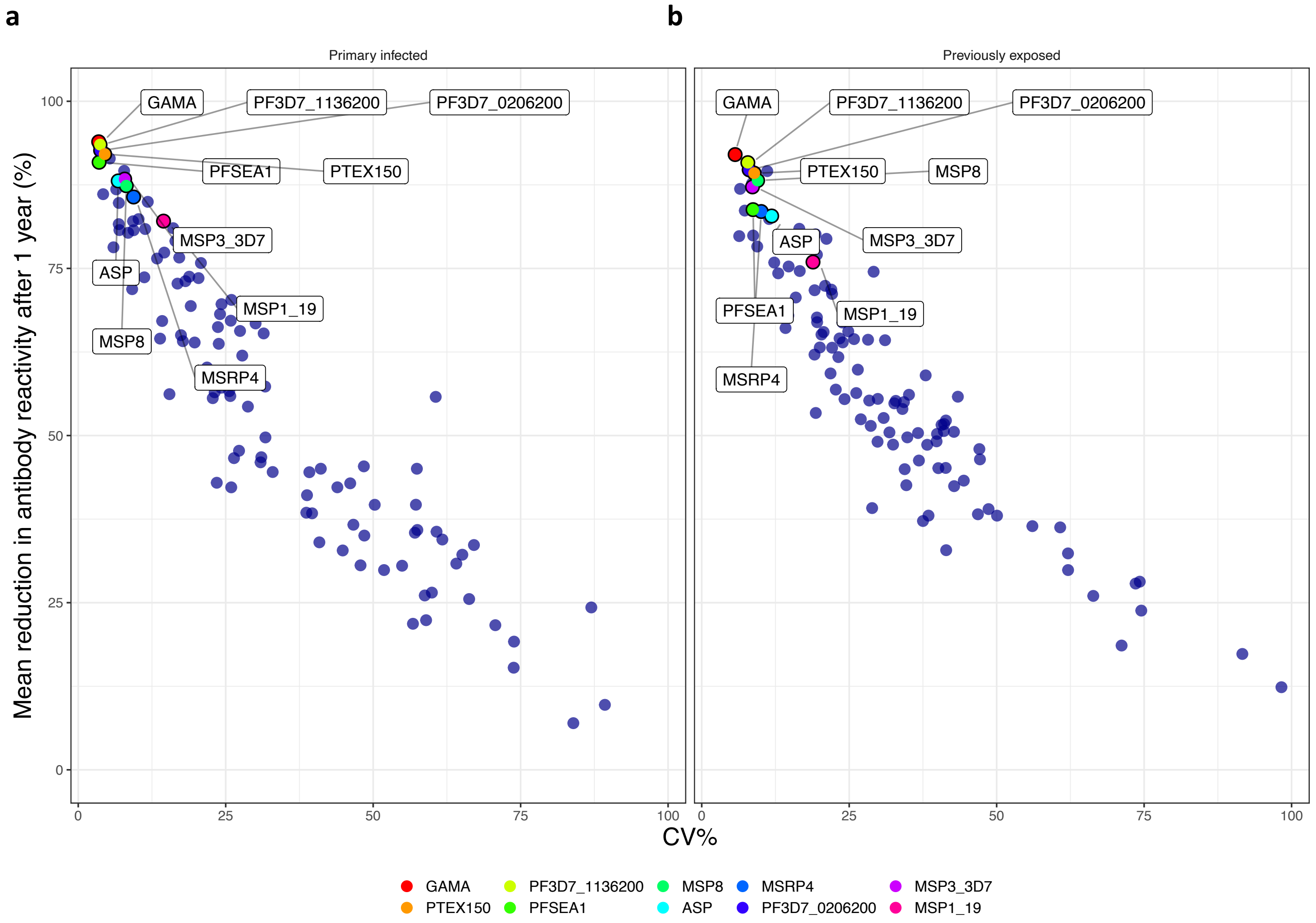

Mean relative reduction in antibody levels after 1 year of follow-up versus the coefficient of variation of the estimated relative reduction in (a) primary infected and (b) previously exposed individuals, respectively. Rainbow colours indicate the antibody responses identified as top 10 most informative in detecting recent infection based on a threshold antibody level to a single antigen.

Supplementary Fig 8

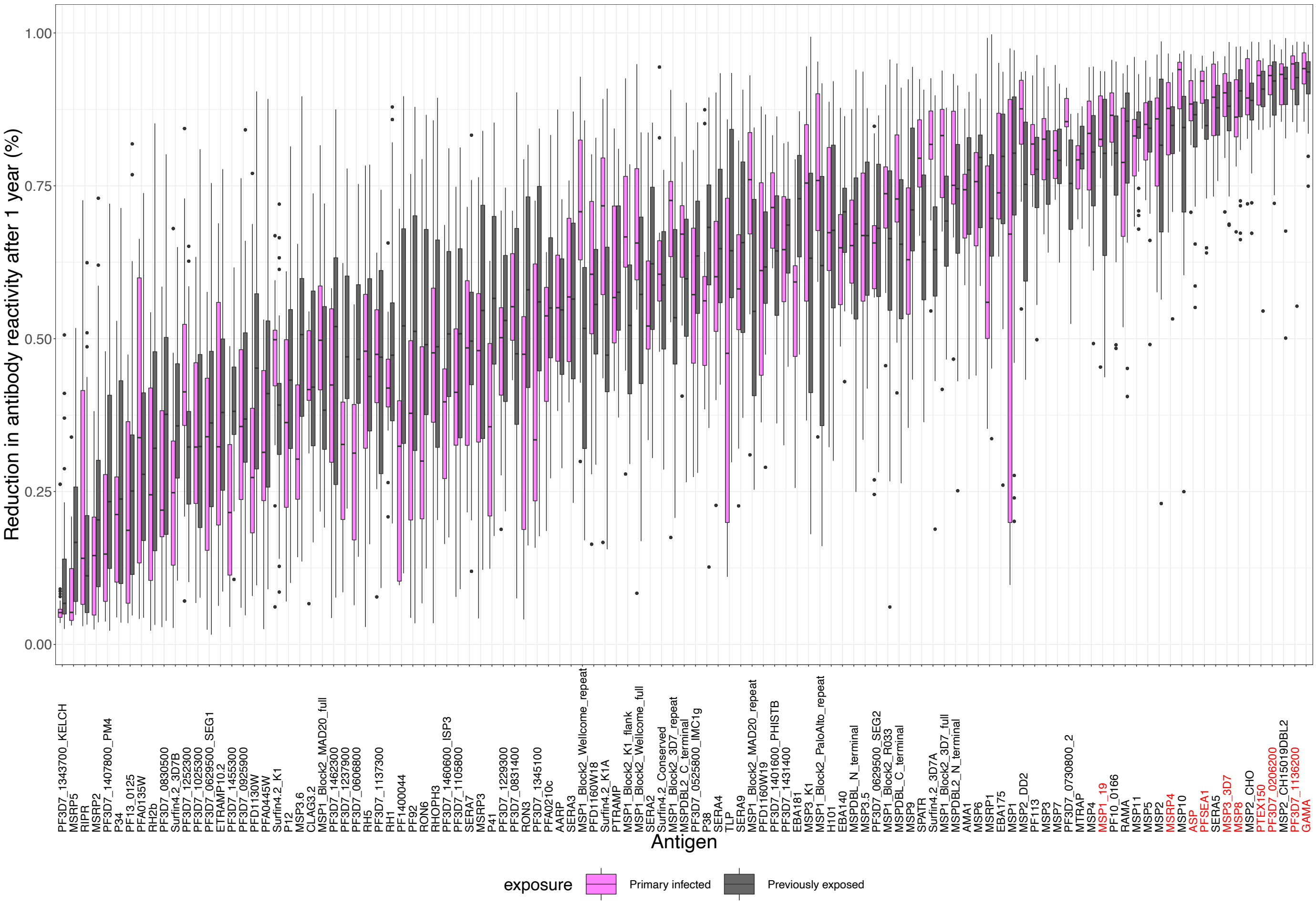

Box-plot of individual antigen-specific antibody kinetic model-estimated relative reduction (%) in antibody levels after one year of follow-up stratified by prior exposure status. The antibody kinetic model was fitted separately to data on each antibody response in 240 longitudinally collected samples from 65 unique individuals. Responses are ordered from left to right by smallest to largest relative reduction in antibody levels. The individual responses identified as top 10 most informative in detecting recent infection based on a threshold antibody level to a single antigen are highlighted in red. Magenta and dark grey boxes indicate data from primary infected individuals and previously exposed individuals, respectively. The centres of boxes correspond to the median. The lower and upper hinges of boxes correspond to the first and third quartiles of the data. The upper and lower whiskers extend from the hinges to the largest and smallest values, respectively, no further than 1.5 * the interquartile range from the hinges. Data beyond the end of the whiskers are plotted individually.
