# Supplementary material for: Distinct kinetics of antibodies to 111 Plasmodium falciparum proteins identifies markers of recent malaria exposure
Source: Nat Commun. 2022 Jan 17;13:331. doi: 10.1038/s41467-021-27863-8 (PMC8764098; doi:10.1038/s41467-021-27863-8)
Supplement: Supplementary file 3 — Description of Additional Supplementary Files [file 41467_2021_27863_MOESM3_ESM.pdf]

Description of Additional Supplementary Files

File Name: Supplementary Data 1
Description: Results from linear mixed effects models examining the difference in antibody levels between primary infected and previously exposed travellers. All antigens and time-points for which antibody levels were significantly different between the two exposure groups are presented within the table. The table contains the following columns: t_id – Sampling time-point identifier; fold_diff – Fold-difference in antibody levels in previously exposed individuals relative to primary infected individuals; qval – False discovery rate (FDR)-adjusted *P*-value.

File Name: Supplementary Data 2
Description: Results from ROC analysis of identifying recent exposure (within 3 months) in travellers based on a threshold antibody level for all individual antigens. The table contains the following columns: auc –Area under the ROC curve (AUC); auc_lb – Lower bound of the 95% confidence interval (CI) of the AUC; auc_ub – Upper bound of the 95% CI of the AUC; roc_sens – Classifier sensitivity at the point of minimal trade-off between sensitivity and specificity; roc_spec – Classifier specificity at the point of minimal trade-off between sensitivity and specificity.

File Name: Supplementary Data 3
Description: The antibody kinetic model estimated population-level parameters and corresponding variance parameters. The table contains the following parameter estimates: Ab_backg – Background antibody reactivity; mu_beta_exp – Mean boost to antibody secreting cells (ASCs) in previously exposed individuals; mu_beta_unexp – Mean boost to ASCs in primary infected individuals; d_cs – Mean half-life of short-lived ASCs (days); d_cl – Mean half-life of long-lived ASCs (days); d_a –Mean half-life of IgG antibodies (days); rho_exp – Mean proportion of new long-lived ASCs in previously exposed participants; rho_unexp – Mean proportion of new long-lived ASCs in primary infected participants; sig_* – Corresponding variance parameters with 95% Credible Interval (CrI). *_low and *_ high represent the lower and upper 95% CrI of the estimated parameters.

File Name: Supplementary Data 4
Description: The antibody kinetic model estimated individual-level parameters. The table contains the following parameter estimates: *_Ab_back – Background antibody reacitivity; *_AB_0 – Mean antibody level at baseline in previously exposed individuals (estimated separately for each individual); *_tau_0 – Time days before presentation to hospital at which antibody levels start to rise (estimated separately for each individual); *_beta – Boost to antibody secreting cells (ASCs); *_t_short – Half-life of short-lived ASCs (days); *_t_long – Half-life of long-lived ASCs (days); *_t_IgG – Half-life of IgG antibodies. *_med, *_low and *_ high represent the median, and lower and upper 95% credible intervals of the estimated parameters, respectively.

File Name: Supplementary Data 5
Description: The antibody kinetic model estimated individual-level peak antibody levels. Values in table correspond to antibody kinetic model estimated individual-level peak antibody levels for all antigens.

File Name: Supplementary Data 6
Description: The antibody kinetic model estimated individual-level relative reduction (%) in antibody reactivity over one year. Values in table correspond to antibody kinetic model estimated individual-level relative reduction (%) in antibody levels over one year.

File Name: Supplementary Data 7
Description: Results from beta regression models examining the association between the relative reduction (%) in antibody levels over 1 year and peak antibody levels, prior exposure status. Regression models were fitted independently to data for all individual antibody responses. The table includes: est – Estimated regression coefficients and the precision parameter for each model; stde – Standard errors of regression parameters; pval – Corresponding *P*-values; qval – False discovery rate (FDR)-adjusted *P*-values. *P*-values were FDR-adjusted for multiple comparisons using the procedure described by Benjamini and Hochberg.

File Name: Supplementary Data 8
Description: Results from ROC analysis of identifying recent clinical malaria (within 3 months) in Kenyan children based on a threshold antibody level for all individual antigens. The table includes: auc – Area under the ROC curve (AUC); auc_lb – Lower bound of the 95% confidence interval (CI) of the AUC; auc_ub – Upper bound of the 95% CI of the AUC; roc_sens – Classifier sensitivity at the point of minimal trade-off between sensitivity and specificity; roc_spec – Classifier specificity at the point of minimal trade-off between sensitivity and specificity.

File Name: Supplementary Data 9
Description: Results from linear regression models examining the association between the magnitude of the antibody response and current infection status and time since last documented clinical malaria episode in Kenyan children. Regression models were fitted independently to data for all individual antibody responses. The table includes: t_cat – ; est – Estimated regression coefficients for each model; ci_lb – Lower bound of 95% confidence interval (CI) of parameter estimate; ci_ub – Upper bound of 95% CI of parameter estimate; pval – Corresponding *P*-values; qval – False discovery rate (FDR)-adjusted *P*-values. *P*-values were FDR-adjusted for multiple comparisons using the procedure described by Benjamini and Hochberg.

File Name: Supplementary Data 10
Description: Data set including antibody data and meta data for the travellers and negative controls. The data set includes the following variables: study_idn – Participant study id; idn – Numeric study id; sample_id – Unique sample identifier; exposure – Previous exposure status; years_in_endemic – Time of residency in endemic area (years); years_since_endemic – Time since residency in endemic area (years); t_id – Time-point id; t_d – Time since malaria diagnosis (days); t_bin – Sample collected within 90 days of diagnosis; antigen – Antigen name; MFI – Antibody reactivity (MFI); log_MFI – $\log_e$ (antibody reactivity); sero_thr – Seropositivity threshold; sero_pos – Seropositive sample.
