# Supplementary material for: Distinct kinetics of antibodies to 111 Plasmodium falciparum proteins identifies markers of recent malaria exposure
Source: Nat Commun. 2022 Jan 17;13:331. doi: 10.1038/s41467-021-27863-8 (PMC8764098; doi:10.1038/s41467-021-27863-8)
Supplement: Supplementary file 14 — Reporting Summary [file 41467_2021_27863_MOESM14_ESM.pdf]

nature research

Corresponding author(s): Victor Yman and Anna Färnert

Last updated by author(s): November 29, 2021

Reporting Summary

Nature Research wishes to improve the reproducibility of the work that we publish. This form provides structure for consistency and transparency in reporting. For further information on Nature Research policies, see our Editorial Policies and the Editorial Policy Checklist.

Statistics

For all statistical analyses, confirm that the following items are present in the figure legend, table legend, main text, or Methods section.

n/a	Confirmed	
☐	☒	The exact sample size (*n*) for each experimental group/condition, given as a discrete number and unit of measurement
☐	☒	A statement on whether measurements were taken from distinct samples or whether the same sample was measured repeatedly
☐	☒	The statistical test(s) used AND whether they are one- or two-sided<br>*Only common tests should be described solely by name; describe more complex techniques in the Methods section.*
☐	☒	A description of all covariates tested
☐	☒	A description of any assumptions or corrections, such as tests of normality and adjustment for multiple comparisons
☐	☒	A full description of the statistical parameters including central tendency (e.g. means) or other basic estimates (e.g. regression coefficient) AND variation (e.g. standard deviation) or associated estimates of uncertainty (e.g. confidence intervals)
☐	☒	For null hypothesis testing, the test statistic (e.g. *F*, *t*, *r*) with confidence intervals, effect sizes, degrees of freedom and *P* value noted<br>*Give P values as exact values whenever suitable.*
☐	☒	For Bayesian analysis, information on the choice of priors and Markov chain Monte Carlo settings
☒	☐	For hierarchical and complex designs, identification of the appropriate level for tests and full reporting of outcomes
☐	☒	Estimates of effect sizes (e.g. Cohen's *d*, Pearson's *r*), indicating how they were calculated

Our web collection on statistics for biologists contains articles on many of the points above.

Software and code

Policy information about availability of computer code

Data collection

Data acquisition from developed antibody microarrays was performed using GenePix® Pro 7 software, Molecular Devices.

Data analysis

All data management and analysis was performed using R (v3.4.4, v3.6.1, and v.4.1.1.).

Robust linear models were fitted using package MASS_7.3-54
Linear mixed effects models were fitted using package nlme_3.1-152
Receiver operating characterstics analysis was performed using packages pROC_1.18.0 and ROCR_1.0-11
Feature selection was performed using package Boruta_7.0.0
Random forest algorithms were fitted using package randomForest_4.6-14
Beta regression models were fitted using package betareg_3.1-4

The R code and data for reproducing the analysis of the travellers data sets are publicly available under an MIT license online at https://github.com/ymanvictor/Pfalciparum_sero_sign

For manuscripts utilizing custom algorithms or software that are central to the research but not yet described in published literature, software must be made available to editors and reviewers. We strongly encourage code deposition in a community repository (e.g. GitHub). See the Nature Research guidelines for submitting code & software for further information.

Data

Policy information about availability of data

All manuscripts must include a data availability statement. This statement should provide the following information, where applicable:

- Accession codes, unique identifiers, or web links for publicly available datasets
- A list of figures that have associated raw data
- A description of any restrictions on data availability

Data generated and analysed within this study is included within the supplementary material of this publication. It is also publicly available online at https://github.com/ymanvictor/Pfalciparum_sero_sign

Field-specific reporting

Please select the one below that is the best fit for your research. If you are not sure, read the appropriate sections before making your selection.

☒ Life sciences ☐ Behavioural & social sciences ☐ Ecological, evolutionary & environmental sciences

For a reference copy of the document with all sections, see nature.com/documents/nr-reporting-summary-flat.pdf

Life sciences study design

All studies must disclose on these points even when the disclosure is negative.

Sample size

Study in Swedish travellers: n = 65 (240 samples).
Study in Kenyan children: n = 280 (280 samples).
Malaria unexposed controls: n = 42 (42 samples).

For the study in Swedish travellers the sample size was determined by the number of eligeable individuals presenting with acute Plasmodium falciparum malaria at the Department of Infectious Diseases, Karolinska University Hospital, Stockholm, Sweden, between 2014 and 2017.

The mathematical modelling of data and statistical inference with classification algorithms which constitute the core analytical framework in this study do not permit classical hypothesis testing, so we cannot undertake formal statistical power calculations. Nonetheless, extensive experience of modelling non-linear data on antibody kinetics (White et al. Lancet Inf Dis 2015, White et al. Lancet Inf Dis 2017, and Yman et al. BMC Medicine 2019) has demonstrated that data from a minimum of approximately 20 participants followed longitudinally after an infection is sufficient for statistical inference with robust characterization of uncertainty. Furthermore, the sample size of the study in swedish travellers corresponds to the sample sizes of previous discovery studies of serological markers of P. falciparum exposure.

The Study in Kenyan children was based on archival and samples and data. The sample size was thus limited by the available sample. However, the sample size corresponds to the sample sizes of previous discovery studies of serological markers of P. falciparum exposure.

Data exclusions

No data was excluded from analysis.

Replication

All antibody responses were measured in three replicates and the average of the three replicates was used for analysis. Positive and negative technical control samples were routinely included in all assay runs and the results from these were reproducible.

Internal cross-validation was performed to asses the robustness of the findings in Swedish travellers. An independent study was conducted in Kenyan children to evaluate the reproducibility of the results obtained in travellers. Findings were successfully replicated across both study settings.

Randomization

This study is observational in nature and no intervention or grouping was applied. As a consequence, no randomisation was required and consequently none was done.

Blinding

This study is observational in nature and no intervention or grouping was applied. Thus, no formal blinding was required. Nevertheless, investigators were blinded to sample exposure status throughout the laboratority analysis of antimalarial antibody responses.

Reporting for specific materials, systems and methods

We require information from authors about some types of materials, experimental systems and methods used in many studies. Here, indicate whether each material, system or method listed is relevant to your study. If you are not sure if a list item applies to your research, read the appropriate section before selecting a response.

Materials & experimental systems

n/a	Involved in the study	
☐	☒	Antibodies
☒	☐	Eukaryotic cell lines
☒	☐	Palaeontology and archaeology
☒	☐	Animals and other organisms
☐	☒	Human research participants
☒	☐	Clinical data
☒	☐	Dual use research of concern

Methods

n/a	Involved in the study	
☒	☐	ChIP-seq
☒	☐	Flow cytometry
☒	☐	MRI-based neuroimaging

Antibodies

Antibodies used

The secondary antibody Alexa Fluor® 647 AffiniPure Donkey Anti-Human IgG, Fcγ fragment specific was purchased from Jackson ImmunoResearch (Product code: 709-605-098, 1 mg / mL).

Validation

Only commercially available secondary antibodies were used.

Human research participants

Policy information about studies involving human research participants

Population characteristics

Swedish study, n = 65
Primary infected participants, n = 21; female: 19% ; age: range 21-59 yrs; Cumulative time of residency in endemic area: range 0-3 yrs, Peak parasitaemia: range <0.1-8.0 %.
Previously exposed participants, n = 44; 23% female; age: range 27-70 yrs; Cumulative time of residency in endemic area: range 13-39 yrs; Time since residency in endemic area: range 0-46 yrs; Peak parasitaemia: range <0.1-7.6%.

Kenyan study, n = 280
age <5 yrs, n = 114, female: 51% (currently infected: n = 16, clinical episode within <3 months: n = 31, clinical episode within 3-12 months: n = 19, no clinical episode during follow-up: n = 48).
age 5-12 yrs, n = 166, female: 51% (currently infected: n = 62, clinical episode within <3 months: n = 31, clinical episode within 3-12 months: n = 26, no clinical episode during follow-up: n = 47).

Recruitment

Swedish study
The study was originally designed to investigate acquisition and maintenance of antimalarial immune responses. Study participants were recruited at Karolinska University Hospital in Stockholm, Sweden from 2014 to 2017. Adults with P. falciparum malaria were enrolled at the time of diagnosis and followed prospectively for up to one year with repeated blood sampling. Venous blood samples were collected at the time of enrolment (i.e. at diagnosis) and follow-up samples were collected approximately ten days, and one, three, six, and twelve months after the first sample. In total, 242 samples were collected from 65 participants. Recruitment was performed on weekdays and during office hours only. Additional patients who might have been eligeable for study inclusion but who were diagnosed and admitted during on-call hours were not recruited. This recruitment scheme is not expected to have lead to any systematic biases. Self-selection biasis is believed to be minimal as grouping of study participants into primary infected and previously exposed individuals was introduced post participant inclusion. We believe that our sample is representative of travellers returning to Sweden with P. falciparum infection. Because we cannot fully ascertain that the adult travellers are representative also of a population living in a malaria endemic area we performed a second study in Kenyan children to evaluate the reproducibility of our results from travellers.

Kenyan study
No recruitment of particapants was done. Archival samples and data from year-long longitudinal observational cohort studies of that were conducted over 2007-2008 in Junju, Kilifi district, Kenya. The study population included 280 children of age 1-12 years. All children were continuously monitored for clinical malaria using passive and weekly active surveillance for febrile illness for 12 months prior to sample collection (i.e. from May 2007 until May 2008). Symptomatic individuals were tested for parasitaemia using blood smears and all individuals positive for P. falciparum were treated for malaria according to Kenyan national guidelines. Samples for serological analysis were collected in a cross-sectional bleed at the beginning of the next more intense malaria transmission season in May 2008 (Murungi et al. 2013, Vaccine). We believe that the study site and population is highly representative of many villages in moderate malaria endemic areas in east Africa, one of the intended target settings for implementing serological tools for monitoring malaria exposure.

Ethics oversight

The Swedish study was approved by the Ethical Review Board in Stockholm, Sweden (Dnr 2006/893-31/4 and 2013/550-32/4, 2018/2354-32, 2019-03436).

The Kenyan study was approved by the Kenya Medical Research Institute (KEMRI) National Ethical Review committee.

Note that full information on the approval of the study protocol must also be provided in the manuscript.
